# Image-Based Assessment of Anti-TNF Treatment Outcomes in Pediatric CRMO/CNO: A Single-Center Case Series

**DOI:** 10.3390/children13081041

**Published:** 2026-08-05

**Authors:** Isabelle G. Houston, Mark D. Heitzmann, Rachel C. Robbins, Katie L. Louka, Erkan Demirkaya, Olcay Y. Jones

**Affiliations:** 1 Department of Pediatrics, Walter Reed National Military Medical Center, Bethesda, MD 20889, USA; isabelle.g.houston.mil@health.mil; 2 Department of Pediatrics, Uniformed Services University of the Health Sciences, Bethesda, MD 20814, USA; 3 Department of Radiology, Walter Reed National Military Medical Center, Bethesda, MD 20889, USA; 4 Department of Rheumatology, Walter Reed National Military Medical Center, Bethesda, MD 20889, USA; 5 DHA Joint Pathology Center, Silver Spring, MD 20910, USA; 6 Division of Pediatric Rheumatology, Western University, London, ON N6A 5W9, Canada

**Keywords:** chronic recurrent multifocal osteomyelitis (CRMO), chronic nonbacterial osteomyelitis (CNO), tumor necrosis factor inhibitor (TNFi), adalimumab

## Abstract

**Highlights:**

**What are the main findings?**

- In this small cohort, early TNFi initiation was associated with sustained clinical improvement.
- Longitudinal, site-specific MRI offered objective measures that informed treatment decisions.

**What are the implications of the main findings?**

- TNFi monotherapy was associated with clinical and radiographic improvement and no serious adverse events.
- Longitudinal focal MRI may provide useful objective data to support patient management.

**Abstract:**

**Background/Objectives:** Chronic recurrent multifocal osteomyelitis/chronic nonbacterial osteomyelitis (CRMO/CNO) is an auto-inflammatory bone disease for which optimal treatment and imaging-based monitoring remain incompletely defined. **Methods:** We conducted a single-center retrospective review of nine pediatric patients with CRMO/CNO treated with tumor necrosis factor inhibitors (TNFis). Clinical improvement was assessed using Visual Analog Scale (VAS) pain scores, erythrocyte sedimentation rate (ESR), and physical examination findings. Radiographic assessment used longitudinal, site-specific magnetic resonance imaging (MRI) scored with a simplified approach adapted from the Chronic Nonbacterial Osteomyelitis Magnetic Resonance Imaging Scoring (CROMRIS) system. **Results:** Our cohort was composed of six females and three males with a median age of seven years. TNFi monotherapy was initiated as a first-line therapy (*n* = 4) or step-up (*n* = 5). During a median follow-up time of 26 months, treatment was tolerated well, except for the emergence of psoriasis in three patients (*n* = 3), among whom one necessitated treatment change. Eight patients on TNFis achieved clinical remission, including five with complete resolution of MRI lesions. **Conclusions:** In this small, single-center case series, TNFi monotherapy was associated with sustained disease control, as reflected by clinical improvement and MRI-documented lesion resolution. These findings emphasize the need for validation of our observations in larger multi-center cohorts.

## 1. Introduction

Chronic recurrent multifocal osteomyelitis, also known as chronic nonbacterial osteomyelitis (CRMO/CNO), is a rare auto-inflammatory bone disorder that causes sterile inflammation at single or multiple skeletal sites and often follows a relapsing–remitting course. It occurs at an estimated incidence of 1 per million children [1], most commonly at ages 7 and 12 years [2], and at a female-to-male ratio of 4:1 [3]. Lesions typically involve the metaphyses of long bones, although nearly any skeletal site may be affected. CRMO/CNO can overlap with other auto-inflammatory conditions, including psoriasis, acne fulminans, ankylosing spondylitis (AS), and inflammatory bowel disease (IBD) [4].

CRMO/CNO remains a diagnosis of exclusion because no disease-specific biomarkers are currently available. Diagnosis, therefore, depends on pattern recognition and the systematic exclusion of mimicking conditions. EULAR/ACR classification criteria provide a diagnostic framework with a reported sensitivity and specificity of 82% and 98%, respectively. The criteria require the absence of alternative etiologies and incorporate objective elements, including histopathology, radiologic features, and basic inflammatory markers such as erythrocyte sedimentation rate (ESR) and C-reactive protein (CRP) [5]. MRI has become particularly important and can often obviate the need for bone biopsy in selected patients [6]. Imaging is also indispensable for assessing disease activity and is incorporated into composite activity measures [7,8,9,10]. More recently, whole-body MRI (WBMRI) has been increasingly favored given the multifocal nature of CRMO/CNO, which often includes symmetric disease distribution [11,12,13]. WBMRI permits the detection and standardized evaluation of affected sites using emerging tools such as the Chronic Nonbacterial Osteomyelitis Magnetic Resonance Imaging Scoring (CROMRIS) system [14]. Despite its utility, longitudinal MRI-based monitoring of treatment response remains limited, largely because of health-system cost constraints and access barriers.

No therapies are approved by the U.S. Food and Drug Administration specifically for CRMO/CNO, and management relies largely on the off-label use of antirheumatic agents, often in a trial-and-error manner. Current consensus-based protocols generally endorse a stepwise treatment strategy: nonsteroidal anti-inflammatory drugs (NSAIDs) as a first-line therapy, followed, when needed, by methotrexate, bisphosphonates, or biologic disease-modifying antirheumatic drugs (bDMARDs), such as TNFis, or a combination of these agents [15,16]. In practice, TNFi therapy is typically reserved for refractory disease, in part because of cost and access limitations [15]. Several retrospective reports have described encouraging safety and efficacy outcomes with TNFi therapy for CRMO/CNO. We present a single-center, real-world experience of treatment response in children with CRMO/CNO receiving TNFis, assessed using longitudinal MRI evaluations.

## 2. Materials and Methods

This retrospective case series was conducted in accordance with Institutional Review Board protocol EDO-20200493 and included patients followed in the Pediatric Rheumatology Clinic at Walter Reed National Military Medical Center. Inclusion criteria were a diagnosis of CRMO/CNO based on EULAR/ACR classification criteria [5] and treatment with a TNFi.

Treatment response was assessed using VAS pain scores, ESR, physical examination findings, and longitudinal, site-specific MRI. Image analysis was performed in an unblinded manner by a senior staff pediatric radiologist (MDH) using an abbreviated scoring system adapted from CROMRIS. The scoring system focused on bone marrow and soft-tissue/periosteal hyperintensity. Bone marrow hyperintensity was graded on a scale from 0 to 2 (0, absent; 1, less than fluid signal intensity; 2, near-fluid signal intensity), and soft-tissue/periosteal hyperintensity was graded on a scale from 0 to 1 (0, absent; 1, present). These metrics were combined to produce a composite abbreviated disease hyperintensity score ranging from 0 to 3. Components associated with chronic or structural disease, such as periosteal reaction, hyperostosis, and physeal changes, were excluded to focus on findings associated with acute inflammation.

Treatment outcomes were evaluated across three domains: (1) clinical remission, defined as the absence of symptoms and a VAS score of 0, irrespective of MRI findings; (2) radiographic remission, defined as complete resolution of MRI abnormalities; and (3) complete remission, defined as simultaneous achievement of clinical and radiographic remission for at least 6 consecutive months.

Serious adverse events (SAEs) associated with TNFi therapy were classified in accordance with Common Terminology Criteria for Adverse Events (CTCAE) version 6.0 and included: (1) serious infections, including tuberculosis, bacterial sepsis, invasive fungal infections, and other opportunistic infections; (2) malignancies, including lymphoma and hepatosplenic T-cell lymphoma, as highlighted in the FDA boxed warning for children and adolescents treated with TNF blockers; (3) serious injection-site reactions, including anaphylactoid reactions; (4) new-onset autoimmune phenomena, including drug-induced lupus-like syndrome and paradoxical psoriasis requiring inpatient management; (5) demyelinating disorders of the central or peripheral nervous system; (6) hematologic abnormalities, including cytopenias; and (7) hepatotoxicity.

## 3. Results

### Findings

Nine patients with CRMO/CNO were included from 12 patients referred to our clinic. Three patients were excluded because of inadequate follow-up and/or a lack of TNFi therapy. As shown in Table 1, six patients were female and three were male. The median age was 7 years at diagnosis and 8 years at the initial rheumatology visit. Common clinical findings included point tenderness and pain at the affected site without evidence of arthritis. The median VAS pain score before TNFi initiation was 8. Two patients (22.2%) had recent-onset fever, one (11.1%) had prominent lymphadenopathy, and four (44.4%) had an elevated ESR (Table 1).

**Figure 1:**
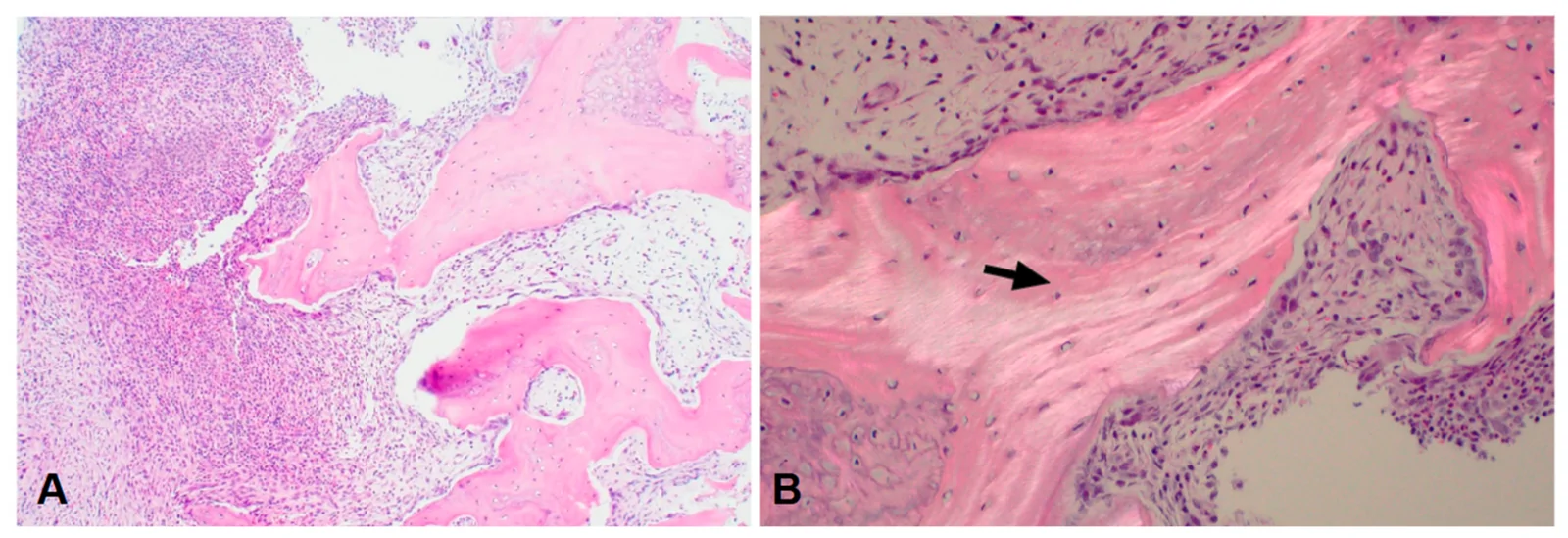
Bone biopsy histopathology from Patient 3. (**A**) 10× magnification: fibrotic marrow with mixed acute and chronic inflammation. Lamellar bone exhibits irregular borders, accelerated osteoclastic and osteoblastic activity and focal tunneling. (**B**) Polarized view, 20× magnification: highlighted lamellae (arrow). Uncoupled bone remodeling disrupts normal sequential deposition, resulting in irregular osteoid edges.

**Figure 2:**
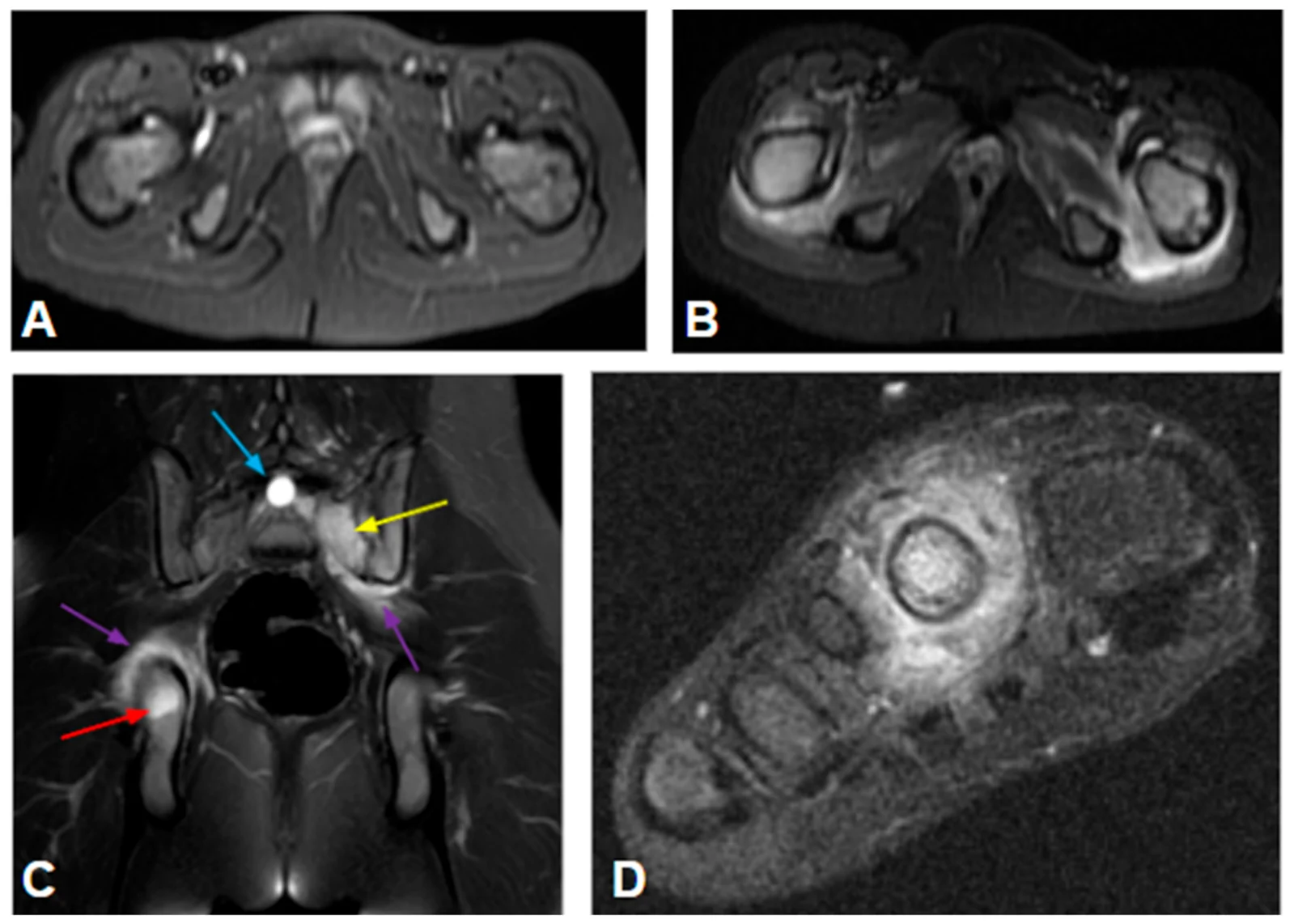
Pelvic and extremity magnetic resonance imaging (MRI) findings before and after treatment. (**A**) Patient 2; axial STIR image of the pelvis at 26 months of TNFi therapy: follow-up imaging demonstrates near-complete resolution of the prior BMH, with bilateral proximal femoral signals matching normal background bone marrow (ischial tuberosities and pubic symphysis), along with resolved soft- tissue hyperintensity. (**B**) Patient 2; axial short-tau inversion recovery (STIR) image of the pelvis prior to tumor necrosis factor inhibitor (TNFi) initiation: Marked bilateral proximal femoral bone marrow hyperintensity (BMH) tracking with fluid-equivalent signal intensity accompanied by extensive surrounding soft -tissue and muscular edema. (**C**) Patient 9; coronal T2 fat-saturated image of the pelvis: marked BMH within the right ischium (red arrow) demonstrating fluid-equivalent signal intensity (blue arrow), and moderate BMH within the left sacrum (yellow arrow) measuring below fluid signal. Surrounding soft-tissue edema is present (purple arrows). (**D**) Patient 1; coronal STIR image of the right foot prior to TNFi initiation: intense BMH within the second metatarsal demonstrating fluid-equivalent signal, with marked surrounding soft-tissue edema.

The initial workup included bone biopsy in seven patients and MRI in all nine patients. As depicted in Figure 1, common histopathologic findings included acute and chronic inflammatory changes in the marrow and evidence of osteoclast activation within bone. Initial MRI assessment revealed bone marrow hyperintensity (BMH) and surrounding soft-tissue edema, as exemplified in Figure 2A,C,D. The median initial abbreviated disease hyperintensity score was 3 on a scale from 0 to 3, with higher scores indicating greater inflammatory activity.

As summarized in Table 1, the median interval from diagnosis to TNFi initiation was 0.83 years. Before TNFis, four patients had failed NSAID therapy as a first-line treatment; two of these patients subsequently failed pamidronate and/or methotrexate (Patients 8 and 9). One patient failed methotrexate as a first-line therapy (Patient 4). All nine patients started adalimumab at a dose of 20 or 40 mg subcutaneously every 2 weeks based on body weight. The dose and/or dosing frequency was temporarily adjusted in some patients, up to 40 mg once weekly, to optimize treatment response until the achievement of clinical improvement.

During follow-up, eight patients (88.9%) showed clinical remission during TNFi therapy based on VAS pain scores, physical examination findings, and ESR. Improvement occurred sequentially: fever resolved almost immediately, pain resolved within a median of 6 weeks (range, 3–24 weeks), and daily activities were gradually regained over 3–6 months.

Longitudinal MRI scores generally correlated with clinical improvement. Five patients (Patients 1, 2, 3, 4, and 6) were full responders to TNFis and achieved an MRI score of 0 after a median of 26 months of TNFi treatment, generally following a monophasic course. As shown in Figure 2, these patients demonstrated normalization of T2 signal intensity, complete resolution of the lesions, a reduction in lesion measurements to 0, and normalization of the contour of the affected bone. Two patients (Patients 7 and 8) had partial improvement, with final MRI scores of 1. Patient 7, who had a large clavicular lesion, achieved clinical remission and had improved examination findings while continuing treatment. Patient 8 was able to wean off treatment after 38 months based on clinical remission; repeat MRI was not available. Of the remaining two patients, Patient 5 achieved an MRI score of 1 at 32 months of therapy, from an initial score of 3, but experienced a flare after a 3-month treatment interruption in the setting of consecutive respiratory infections. Patient 9 had a protracted course that required a change in medication, as detailed below. The time course of MRI scores is summarized in Supplementary Figure S1.

Overall, most patients experienced treatment-dependent mini-flares during follow-up. These episodes typically began with localized pain accompanied by MRI changes. Common triggers included attempted dose reduction of adalimumab after clinical remission had been achieved during the COVID-19 pandemic and forced treatment interruption because of an inciting infection. Holding adalimumab for more than 4–6 weeks or increasing the dosing interval from every 2 weeks to every 3–4 weeks resulted in an increased MRI score (Patients 5 and 7) or the emergence of new lesions (Patients 5 and 6). Streptococcal infections were another potential trigger. During follow-up, five patients had culture-confirmed group A streptococcal (GAS) infections with elevated streptococcal serologies. Three of these five patients developed post-streptococcal complications. Patient 6 developed a mini-flare after a streptococcal infection while off adalimumab by choice after 16 months of treatment. Patient 9 experienced disease flares on two separate occasions, both preceded by GAS infections (see below). After recovering from CRMO/CNO, Patient 4 was later diagnosed with pediatric autoimmune neuropsychiatric disorders associated with streptococcal infections (PANDAS) at age 15. All mini-flares resolved within 6–8 weeks after temporary TNFi dose titration.

The most common adverse event was a paradoxical psoriasiform eruption, which occurred in three of nine patients (Patients 3, 8, and 9). Patient 3 developed mild lesions in the setting of high anti-adalimumab antibody titers and worsening MRI findings but improved after switching to etanercept. Patient 8 successfully managed mild lesions with topical therapy while continuing adalimumab. Patient 9 experienced severe progression, including palmoplantar involvement and significant hair loss, which necessitated the discontinuation of adalimumab in favor of alternative DMARDs. One patient (Patient 5) had infection-related concerns that led to a temporary interruption of treatment. No SAE requiring inpatient management was observed.

Patient 9 first presented at 7 years of age after 1 year of treatment with pamidronate (12 cycles at 24 mg/kg). She was subsequently started on adalimumab and developed psoriasis, with severe hair loss within 4 months. During the course of her disease, she developed new spinal lesions in year 3 and hip lesions in year 5 after the CRMO/CNO diagnosis; both episodes followed streptococcal infections. Her treatment was changed to anti-IL-1 biologic agents for a total of 13 months (anakinra for 5 months and canakinumab for 8 months), followed by thalidomide at 50 mg/day 5 years into her disease. She achieved complete remission and was able to taper off thalidomide after 2 years of therapy.

At the most recent assessment, six of nine patients (Patients 1, 2, 3, 4, 6, and 9) were asymptomatic and had normalized MRI findings, meeting the definition of complete remission (66.7%). Five of these six patients achieved complete remission while receiving a TNFi, and one achieved remission while receiving thalidomide (Patient 9). Three patients (Patients 2, 3, and 6) were in remission on treatment, and three (Patients 1, 4, and 9) were in remission off treatment. The remaining three patients (Patients 5, 7, and 8) were clinically asymptomatic, had improved MRI scores, and continued TNFi therapy, meeting the definition of clinical remission. Overall, eight of nine patients achieved either clinical or complete remission during TNFi therapy.

## 4. Discussion

We report a single-center experience involving nine patients with CRMO/CNO treated with TNFis as a first-line therapy (*n* = 4) or after prior therapies including NSAIDS, methotrexate, and/or bisphosphonate therapy (*n* = 5). This exploratory study revealed that eight of nine patients (88.9%) achieved clinical remission while on TNFi therapy. This included five (55.6%) with complete resolution of MRI lesions during a median follow-up time of 26 months. Treatment was generally well tolerated. Paradoxical psoriasis occurred in three patients; two were managed with topical treatment, and one required discontinuation of TNFi therapy.

These findings align with prior retrospective data [17,18,19,20]. Gaal et al. reported a 60% full response rate over 2 years among five patients treated with TNFis, defining response as the resolution of clinical symptoms, normalization of inflammatory markers, and resolution of or minimal residual MRI abnormalities [19]. Similarly, Schnabel et al. described 44 pediatric patients across eight international medical centers who were treated with TNFis after inadequate response to NSAIDs or bisphosphonates, demonstrating clinical remission in 65% and WBMRI recovery in 52% at 12 months [20]. Our observed rates of clinical recovery and paradoxical psoriasiform eruptions during TNFi therapy are broadly consistent with these published cohorts.

Standardized definitions of disease activity and remission in CRMO/CNO remain under refinement [17]. We defined complete remission as the resolution of clinical signs and symptoms together with the absence of active inflammatory lesions on MRI, consistent with prior reports [21]. One limitation of our study is the use of site-specific MRI rather than WBMRI for initial and longitudinal assessment. This approach was largely driven by the absence of pain or other symptoms to justify broader imaging, as well as by challenges related to access to prolonged imaging studies. Although WBMRI is recommended for comprehensive assessment of disease burden [22], serial focal MRI provided high-resolution monitoring of previously active lesions, including in clinically asymptomatic patients. Because CROMRIS has been validated for WBMRI and no standardized scoring system currently exists for focal MRI [14], we adapted a simplified CROMRIS-based approach. Based on our experience, it is reasonable to propose a pragmatic surveillance strategy involving baseline WBMRI at diagnosis followed by interval focal MRI for targeted monitoring of previously active or clinically relevant lesions. Repeat WBMRI can be reserved for inadequate response, suspected flare, or concern for multifocal progression.

Our therapeutic approach incorporated MRI-guided modulation of TNFi dose and dosing frequency, allowing individualized control of inflammation while maintaining biologic monotherapy without adjunctive corticosteroids, conventional DMARDs, or bisphosphonates. Conceptually, this strategy is analogous to corticosteroid titration, a common practice in the management of many rheumatologic diseases. MRI evidence of inflammatory activity guided biologic optimization. Progressive reductions in MRI inflammatory scores aligned with clinical improvement, suggesting the value of an imaging-integrated treatment framework.

The rationale for prioritizing TNFis is grounded in the pathogenesis of CRMO/CNO. Histopathologic and translational data implicate myelomonocytic infiltration and macrophage-driven cytokine networks in sustaining sterile osteitis. Activation of the NFkB pathway and upregulation of TNF-α represent a central upstream mechanism affecting both circulating and tissue-resident macrophages [23,24,25,26]. TNFis, therefore, provide a pathway-specific treatment modality to attenuate the auto-inflammatory axis. The clinical responsiveness to bisphosphonates [27] also underscores the contribution of osteoclast-mediated bone remodeling; however, whether osteoclast activation is primary or secondary to cytokine-driven inflammation remains unresolved.

Two observations notable as hypothesis-generating insights included CRMO/CNO flares (Patients 6 and 9) temporally associated with exposure to group A β-hemolytic Streptococcus-hemolytic *Streptococcus* (GABHS). Although, to our knowledge, this is an uncommon sequelae of GABHS, microbial-derived subcellular components—such as pore-forming toxins known to induce NLRP3 activation [28]—could act as inflammatory amplifiers despite the sterile nature of CRMO/CNO. Notably, susceptibility appeared to be host- or strain-specific, as one patient developed PANDAS without a concurrent CRMO/CNO flare. Second, we observed mild-to-moderate paradoxical psoriasis among three patients during TNFi therapy that was managed in the outpatient setting without inpatient admission. These events may reflect complex cytokine feedback mechanisms within osseous inflammatory niches. One patient with incomplete response to TNFi and IL-1 blockade achieved remission with thalidomide, which highlights mechanistic heterogeneity and the potential need for alternative pathway modulation in refractory disease. Thalidomide, used as a novel approach, is known to downregulate a number of pro-inflammatory cytokines [29], which cautiously justifies its off-label application.

Several limitations inherent to our design merit discussion. First, the small cohort (*n* = 9) limits statistical power, precludes formal hypothesis testing, and prevents identification of baseline predictors of treatment response. The findings should, therefore, be interpreted as trend-indicating rather than definitive. Second, the absence of a concurrent control or conventional treatment comparison group prevents definitive conclusions regarding the superior efficacy of TNFis over standard therapies. Furthermore, because only four patients were treatment-naïve, prior exposure to NSAIDs, methotrexate, or pamidronate by initial providers confounds the evaluation of TNFis as a strict first-line therapy. The observed favorable outcomes may partially reflect our patient population’s access to care and opportunity for early intervention that may provide an advantage over civilian healthcare and influence TNFi efficacy. As a retrospective, single-center study, our findings are subject to selection bias and have limited external validity compared with the broader and more heterogeneous pediatric CRMO/CNO population. Lastly, our radiographic outcomes were monitored using a simplified, site-specific scoring system adapted from CROMRIS components. While this approach was necessary due to the localized nature of the available clinical imaging, this modified scoring system lacks formal external validation. Consequently, our radiographic trends should be interpreted as localized markers of inflammatory volume rather than validated whole-body structural scores.

In conclusion, CRMO/CNO remains a challenging pediatric condition with limited treatment options. Our observations on these nine patients are promising and warrant further validation.

## Figures and Tables

**Table 1:** Summary of nine patients in the study cohort.

Parameter	1	2	3	4	5	6	7	8	9
**Age at Dx**	12	3	6	12	6	13	7	8	7
**Sex**	F	F	F	M	M	M	F	F	F
**Bone Biopsy**	No	No	Yes	Yes	Yes	Yes	Yes	Yes	Yes
**Initial ESR**	41	92	21	69	11	15	44	3	28
**Prior Therapies**	None	None	None	MTX	None	NSAIDs	NSAIDs	NSAIDs Pamidronate	NSAIDs MTX Pamidronate
**Age 1st TNFi**	12	3	7	13	7	14	9	11	10
**Current Treatment**	None	Adalimumab	Etanercept	None	Adalimumab	Adalimumab	Adalimumab	None	None
**CRMO Site**	Ankle	Femurs	Mandible	Foot	Tibia	Ulna	Clavicle	Mandible	Clavicle Spine Pelvis
**Initial MRI Score**	2	3	1	3	3	2	2	3	3
**Last MRI Score**	0	0	0	0	1	0	1	1	0
Time to Remission *	2.17	2.17	1.42	4	NA	0.83	NA	NA **	4.7 ***
**Follow up time**	10	3	4	9	7	4	4	8	15

Age, time to remission, and follow-up time are presented in years. Abbreviations: ESR, erythrocyte sedimentation rate (normal range: less than 20 mm/h); F, female; M, male; MRI, magnetic resonance imaging; MTX, methotrexate; NA, not applicable (patient did not achieve complete remission per MRI); NSAIDs, nonsteroidal anti-inflammatory drugs. Notes: MRI scores are reported for the most prominent focal lesion over time. * refers to complete remission. ** indicates the patient relocated. *** indicates the patient achieved remission on thalidomide.

## Data Availability

The data presented in this study are available on request from the corresponding author due to privacy and ethical restrictions regarding human participant confidentiality.

## Supplementary Materials

The following supporting information can be downloaded at: https://www.mdpi.com/article/10.3390/children13081041/s1, Figure S1. Longitudinal changes in MRI scores over time. The x-axis displays time from treatment initiation plotted on a logarithmic scale (log years) to visualize rapid early radiographic shifts alongside long-term follow-up. The y-axis represents the localized MRI severity score, with lower values indicating a reduction in inflammatory volume and radiographic resolution.

## Abbreviations

The following abbreviations are used in this manuscript:

CRMO	Chronic recurrent multifocal osteomyelitis
CNO	Chronic nonbacterial osteomyelitis
GABHS	β-hemolytic Streptococcus-hemolytic *Streptococcus*
SAE	Serious adverse event
STIR	Short-tau inversion recovery
TNFi	Tumor necrosis factor inhibitor
WBMRI	Whole-body MRI
