# Supplementary material for: Image-Based Assessment of Anti-TNF Treatment Outcomes in Pediatric CRMO/CNO: A Single-Center Case Series
Source: Children (Basel). 2026 Aug 5;13(8):1041. doi: 10.3390/children13081041 (PMC13511615; doi:10.3390/children13081041)
Supplement: Supplementary file 1 [file children-13-01041-s001.zip › children-4406291-supplementary.pdf]

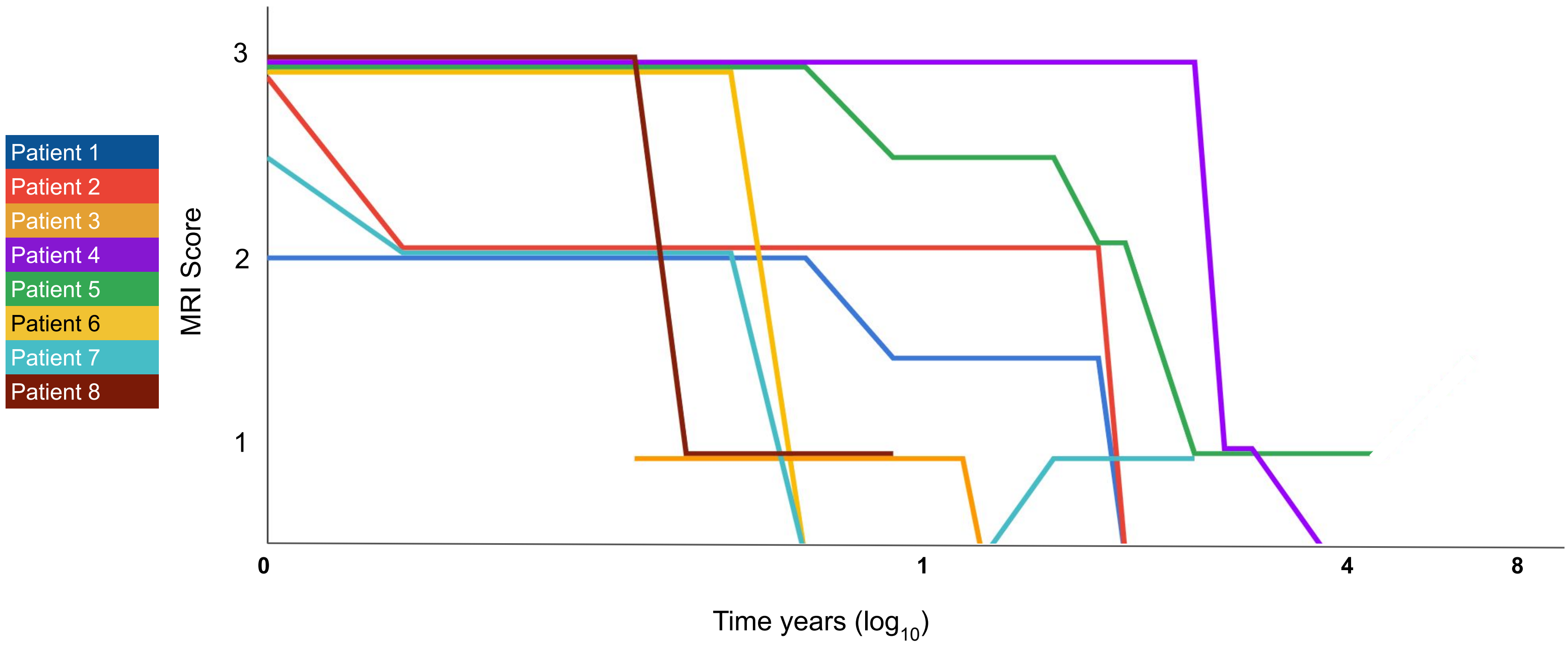

Supplementary Figure S1: Longitudinal changes in MRI scores over time. The x-axis displays time from treatment initiation plotted on a logarithmic scale (log years) to visualize rapid early radiographic shifts alongside long-term follow-up. The y-axis represents the localized MRI severity score, with lower values indicating a reduction in inflammatory volume and radiographic resolution.
